# Effects of Parathyroidectomy on Muscle Mass, Strength, and Physical Function in Uremia-Associated Secondary Hyperparathyroidism

**DOI:** 10.3390/healthcare14162488

**Published:** 2026-08-11

**Authors:** Lingfeng Cai, Mingxia Xiong, Dandan Sheng, Weichun He

**Affiliations:** 1 The Second Clinical Medical Department, Nanjing Medical University, Nanjing 211166, China; 2 Department of Radiology, The Second Affiliated Hospital of Nanjing Medical University, Nanjing 210011, China

**Keywords:** uremia, secondary hyperparathyroidism, parathyroidectomy, muscle mass, muscle strength, motor function

## Abstract

**Objective**: To investigate the effects of parathyroidectomy (PTX) on muscle mass, muscle strength, and motor function at different postoperative stages in uremic patients with secondary hyperparathyroidism (SHPT). **Methods**: A total of 47 uremic patients with SHPT who underwent PTX were enrolled in this study. General clinical data, including age and blood biochemical parameters, were collected. Pre- and postoperative measurements of lean body mass, the appendicular skeletal muscle index (ASMI), skinfold thickness, grip strength, and 6-m walk time were recorded. Patients were divided into three groups—short-term, mid-term, and long-term—based on the timing of postoperative follow-up, and the patterns of change in muscle mass, strength, and function at different time points following PTX were analyzed. **Results**: In the short-term postoperative period, patients’ grip strength increased significantly, and their 6-m walk time decreased significantly (*p* < 0.05), whilst lean body mass, the ASMI and skinfold thickness showed no significant changes compared with preoperative levels (*p* > 0.05). In the mid-term postoperative period, lean body mass, the ASMI and grip strength all increased significantly, whilst the 6-m walk time decreased significantly (*p* < 0.05); there was no significant change in skinfold thickness (*p* > 0.05). In the long-term postoperative period, skinfold thickness and grip strength increased significantly, and the 6-m walk time was significantly reduced (*p* < 0.05), whilst lean body mass and the ASMI showed no statistically significant difference compared to preoperative levels (*p* > 0.05). **Conclusions**: PTX can effectively improve muscle strength and lower limb motor function in uremic patients with SHPT, and these improvements persist during long-term postoperative follow-up; improvements in muscle mass-related indicators exhibit a phased pattern, with significant increases observed in the mid-postoperative period, suggesting that the improvement in muscle mass following PTX is subject to a certain time-dependent effect.

## 1. Introduction

Sarcopenia is a progressive skeletal muscle disorder characterized by reduced skeletal muscle mass, decreased muscle strength, and impaired physical function [1]. Recent studies have confirmed that the prevalence of sarcopenia is significantly higher among uremic patients and that it is an important independent risk factor affecting patient prognosis [2,3,4]. Compared to the general population, uremic patients are at a high risk for sarcopenia due to various pathophysiological factors, including long-term retention of uremic toxins, a state of low-grade inflammation, and metabolic disorders [5,6]. This risk is particularly elevated in patients with secondary hyperparathyroidism (SHPT). Elevated parathyroid hormone (PTH) is associated with adverse changes in skeletal muscle cells. It inhibits muscle protein synthesis and accelerates protein degradation through multiple mechanisms, such as inducing the browning of white adipose tissue to increase whole-body energy expenditure and activating the ubiquitin–proteasome system [7]. Many SHPT patients experience a marked decline in muscle strength. In severe cases, patients may have difficulty standing up from a seated position or be unable to stand at all; some exhibit a shuffling gait, which increases the risk of fractures and may even lead to a complete inability to walk. This severely impacts patients’ quality of life and significantly increases mortality rates [8]. Studies have shown that the quality of life of uremic patients significantly improves following successful parathyroidectomy (PTX) [9]. However, the extent of improvement in muscle mass, strength, and function, as well as the time course of these effects, remains unclear [10]. Currently, methods for assessing sarcopenia primarily include anthropometry and imaging techniques. Among these, dual-energy X-ray absorptiometry (DXA) offers high precision and good reproducibility. It can accurately quantify body composition indicators such as skeletal muscle mass and objectively and reliably reflect the severity of sarcopenia in patients, making it one of the key methods for the clinical assessment of sarcopenia [11]. Therefore, this study employed DXA to assess body composition and sarcopenia status in uremic patients with SHPT. We observed changes in muscle mass, muscle strength, and motor function before and after PTX treatment in these patients to investigate the efficacy of PTX in improving sarcopenia in uremic patients with SHPT.

## 2. Materials and Methods

### 2.1. Study Population

This is a single-center retrospective paired observational study. We consecutively enrolled inpatients who met the inclusion and exclusion criteria at the Department of Nephrology at the Second Affiliated Hospital of Nanjing Medical University between January 2021 and December 2023. Postoperative follow-up was conducted between January 2021 and December 2025. A total of 47 patients with uremia-associated SHPT who were receiving PTX therapy were enrolled in this study. Each patient underwent one round of laboratory and physical examinations at preoperative baseline and one corresponding postoperative test at a single follow-up time point. Based on the timing of their postoperative follow-up visits, patients were divided into three mutually exclusive groups: 14 patients in the short-term group (6–12 months), 26 patients in the mid-term group (12–24 months), and 7 patients in the long-term group (>24 months). SHPT was defined as persistent, excessive parathyroid hormone secretion induced by calcium–phosphorus metabolic disturbance and vitamin D deficiency secondary to uremia, per KDIGO guidelines [12]. Surgical strategies were individualized based on clinical conditions and patients’ willingness, including total parathyroidectomy or total parathyroidectomy combined with autologous parathyroid gland transplantation into the forearm.

Referring to the PTX indications specified in the Chinese expert consensus on surgical practice of secondary hyperparathyroidism associated with chronic kidney disease [13], the inclusion criteria were defined as follows: (1) severe secondary hyperparathyroidism (PTH > 800 pg/mL); (2) hypercalcemia and/or hyperphosphatemia unresponsive to drug therapy (e.g., phosphate-lowering agents, calcitriol pulse therapy) and/or enhanced dialysis; (3) ultrasound confirmation of at least one enlarged parathyroid gland, with at least one having a diameter greater than 1 cm or a volume greater than 0.5 cm^3^; and (4) age 18–70 years.

**Figure 1:**
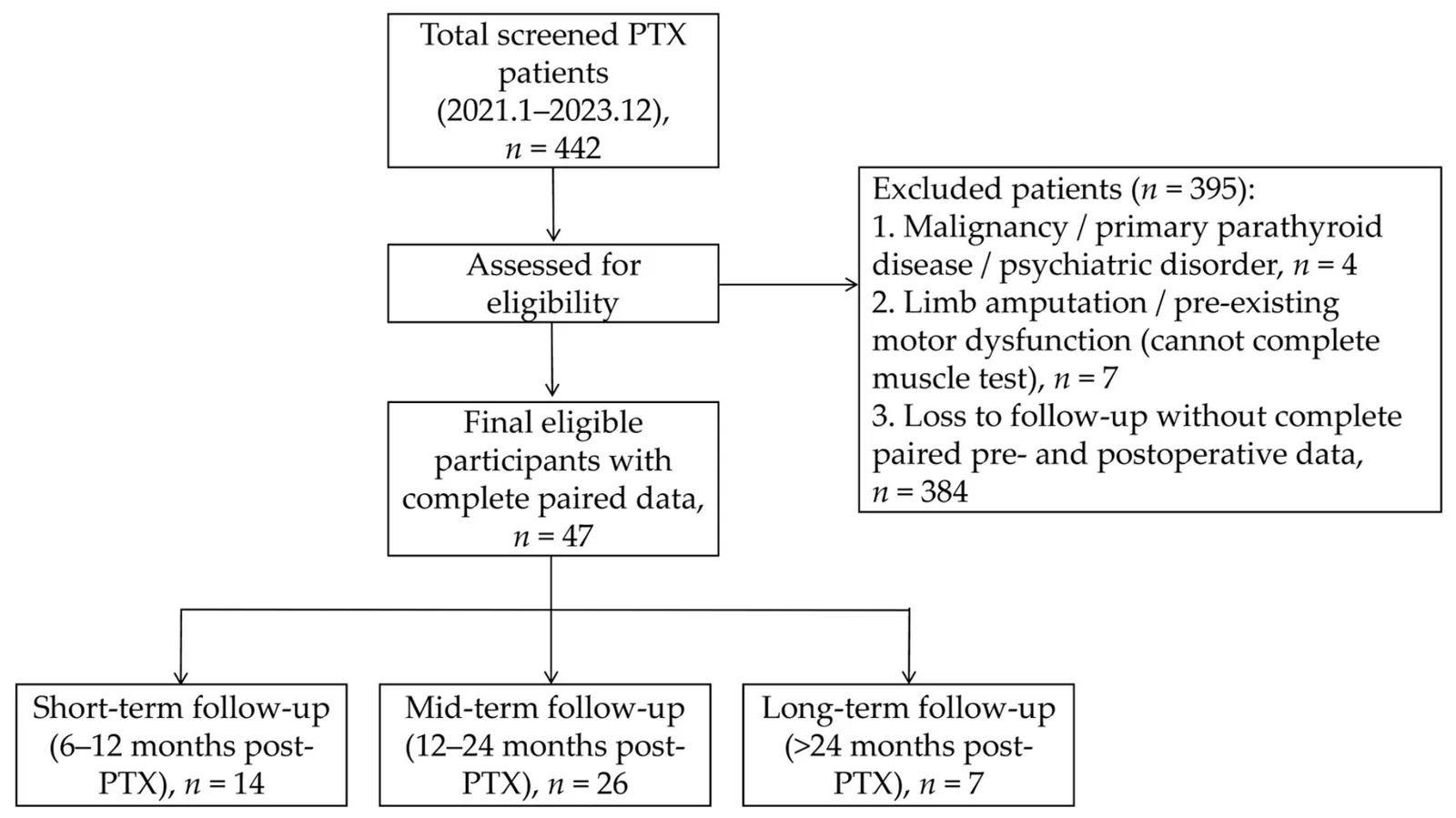
Flow chart of participant selection.

Exclusion criteria were as follows: (1) patients with concurrent malignant tumors, primary parathyroid diseases, or psychiatric disorders; (2) patients with limb amputations, motor dysfunction, or other conditions preventing the completion of muscle function testing; and (3) patients with infections, inflammatory conditions, severe cardiopulmonary diseases, or other conditions rendering them unable to tolerate surgery. All enrolled subjects signed informed consent forms. The participant flow chart is shown (Figure 1).

### 2.2. Collection of General Data

This research was initiated on 27 March 2026. We collected patients’ general demographic and medical information, including: (1) general demographic data—gender, age, duration of dialysis, and dialysis method; (2) anthropometric measurements—height, weight, and body mass index (BMI = weight/height^2^, kg/m^2^); (3) laboratory test results—hemoglobin, myoglobin, troponin, N-terminal pro-B-type natriuretic peptide (NT-proBNP), urea, creatinine (Cr), uric acid, serum phosphorus, serum calcium, bicarbonate, albumin (ALB), total cholesterol (TC), triglycerides (TGs), high-density lipoprotein cholesterol (HDL-C), low-density lipoprotein cholesterol (LDL-C), PTH, alkaline phosphatase (ALP), etc.; and (4) comorbidities and medical history—history of infectious diseases, hypertension, diabetes, coronary heart disease, cerebral infarction, etc. All laboratory values are based on fasting venous blood tests performed after the patient’s admission, conducted in strict accordance with the hospital’s clinical laboratory operating procedures.

### 2.3. Muscle-Related Parameter Testing

Muscle mass, muscle strength, and motor function-related parameters were assessed separately before and after surgery. The specific testing methods are as follows:

Muscle mass parameters: We included lean body mass, appendicular skeletal muscle index (ASMI), and skinfold thickness. Lean body mass was measured using DXA with a Hologic Discovery W dual-energy X-ray absorptiometer (Hologic, Marlborough, MA, USA). ASMI was calculated using body composition analysis and height. Skinfold thickness was measured using a skinfold caliper, and the average of three measurements was used as the final result.

Muscle strength indicators: Grip strength of the patient’s dominant hand was measured using an electronic dynamometer (CAMRY, Zhongshan, Guangdong, China). The patient stood with arms hanging naturally at their sides, palms facing inward, and held the dynamometer with one hand. They squeezed the dynamometer with maximum force in a single attempt. Three measurements were taken, and the mean value was recorded.

Motor function indicators: A 6 m walking test was used. Patients were asked to walk 6 m at a normal pace. Walking time was assessed over three trials, and the mean time was used as the final outcome. Faster walking speed corresponds to shorter walking time, indicating better motor function.

Sarcopenia was diagnosed according to the 2019 consensus of the Asian Working Group for Sarcopenia (AWGS 2019). The corresponding cut-off values are defined as follows: low muscle mass was identified when ASMI measured by DXA was ≤7.0 kg/m^2^ for males and ≤5.4 kg/m^2^ for females; low muscle strength was defined as handgrip strength < 28 kg in males and <18 kg in females; poor physical performance was determined when the 6-m gait speed was less than 1.0 m/s. According to the diagnostic algorithm, sarcopenia was confirmed in patients with low muscle mass combined with low muscle strength or poor physical performance [14].

### 2.4. Statistical Methods

Clinical data were analyzed using SPSS 27.0 software. Normally distributed quantitative data were expressed as mean ± standard deviation. Non-normally distributed quantitative data were presented as medians (25th and 75th percentiles). Categorical data were described as numbers (percentages). For intragroup comparisons between preoperative and postoperative measurements, the paired *t*-test was used for normally distributed data, and the Wilcoxon signed-rank test was applied for non-normally distributed data. For intergroup comparisons among the three subgroups at baseline, a one-way analysis of variance was used for normally distributed quantitative variables, and the Kruskal–Wallis H test for skewed quantitative variables. Intergroup comparisons of categorical variables were conducted using Fisher’s exact test. A *p*-value < 0.05 was considered statistically significant.

## 3. Results

A total of 47 uremic patients with SHPT undergoing parathyroidectomy were included in the analysis. Patients were divided into three groups according to the postoperative follow-up duration. Baseline characteristics are detailed (Table 1), and no significant intergroup differences were observed among subgroups.

### 3.1. Postoperative PTH, Calcium, Phosphorus and ALP Changes

**Figure 2:**
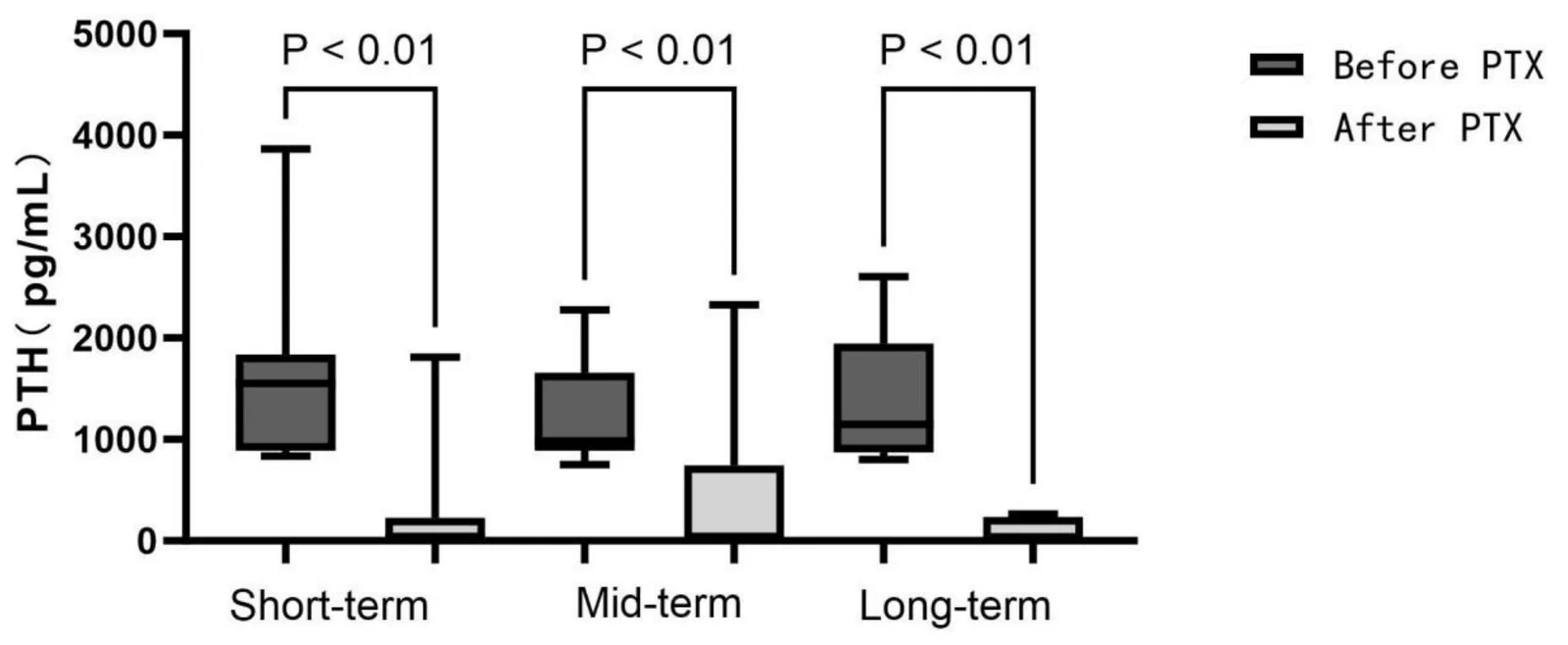
Changes in PTH levels following PTX.

**Figure 3:**
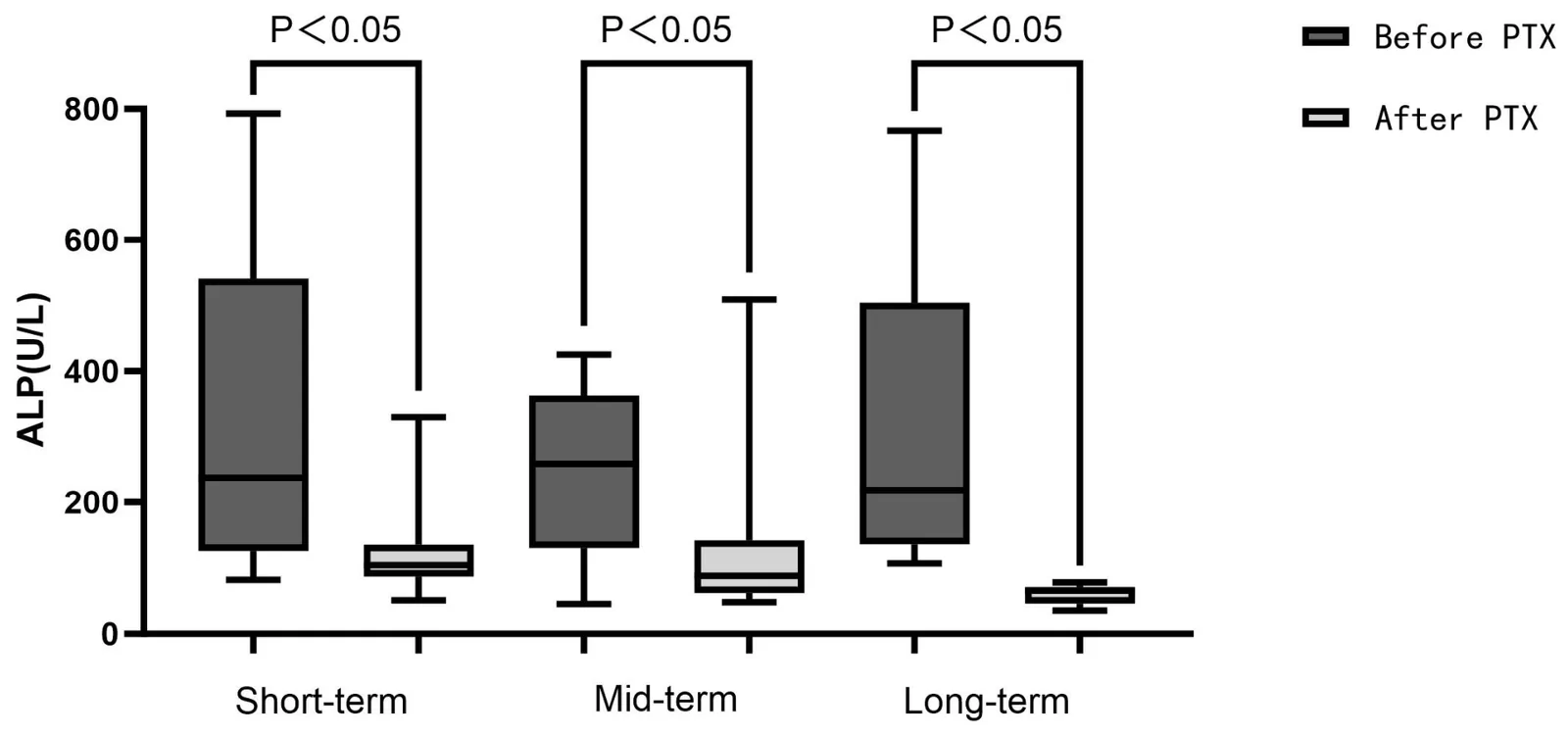
Changes in ALP levels following PTX.

**Figure 4:**
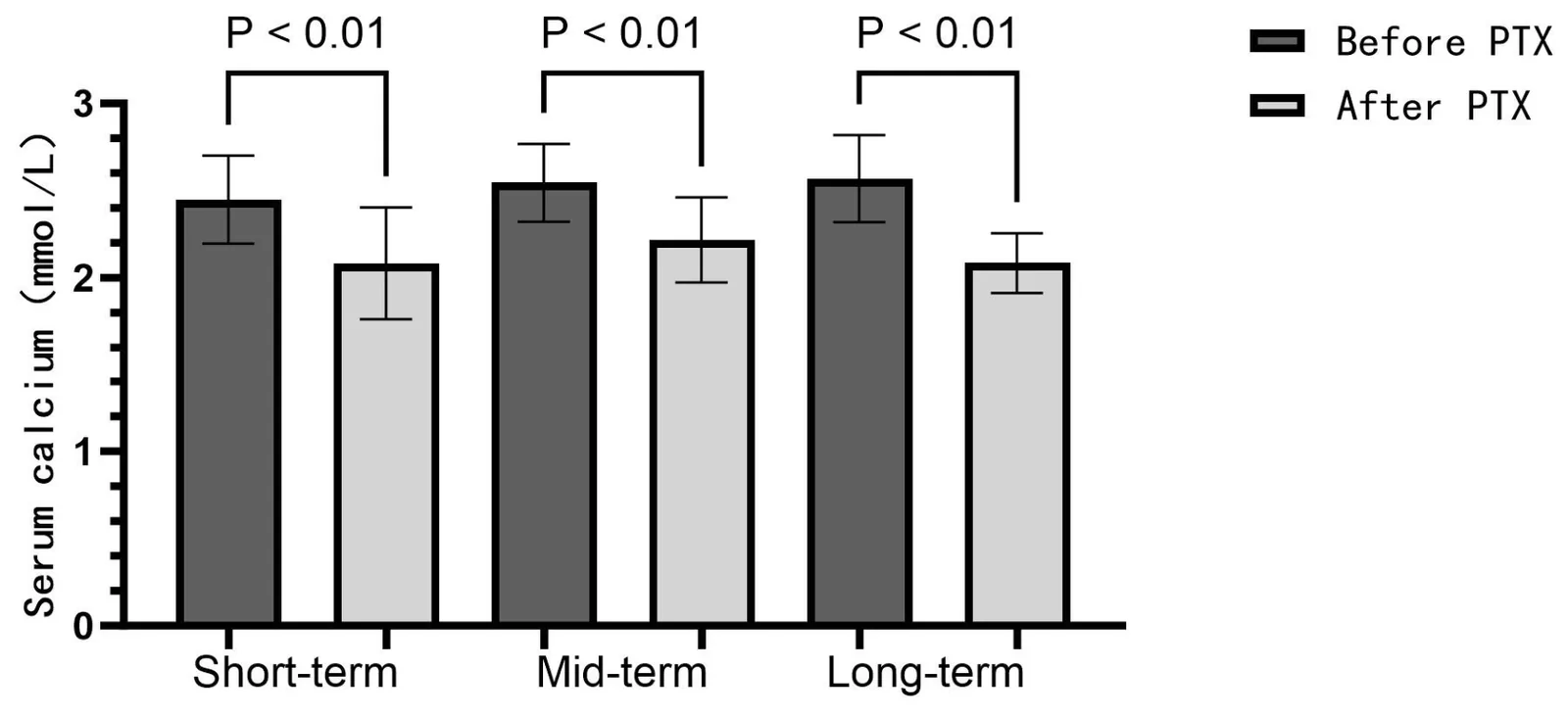
Changes in serum calcium levels following PTX.

**Figure 5:**
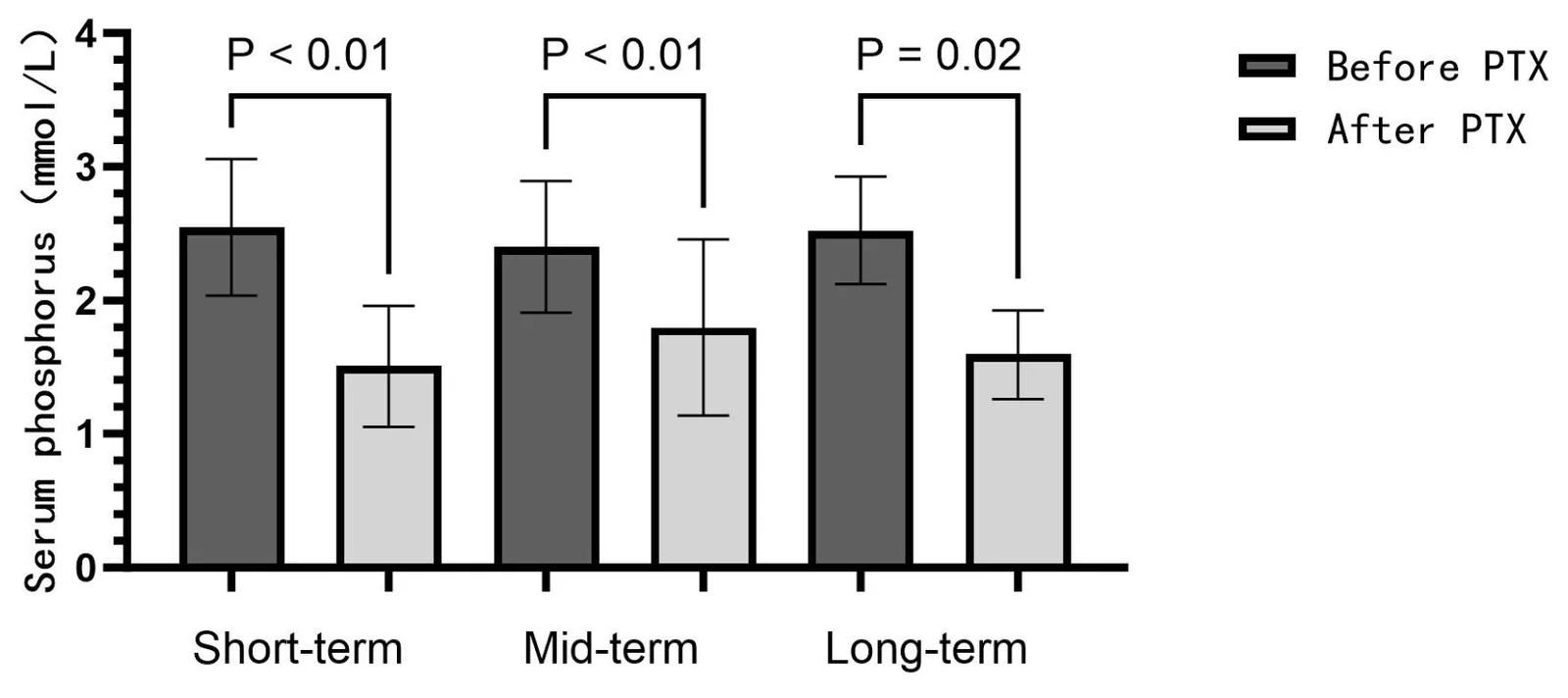
Changes in serum phosphorus levels following PTX.

At all follow-up time points, patients’ postoperative serum PTH, phosphorus, calcium, and ALP levels were significantly lower than preoperative levels, suggesting that surgery effectively alleviates the persistent toxic effects of excessive PTH on muscle metabolism, thereby laying the foundation for muscle function recovery (Figure 2, Figure 3, Figure 4 and Figure 5).

### 3.2. Improvement in Sarcopenia Postoperatively

**Figure 6:**
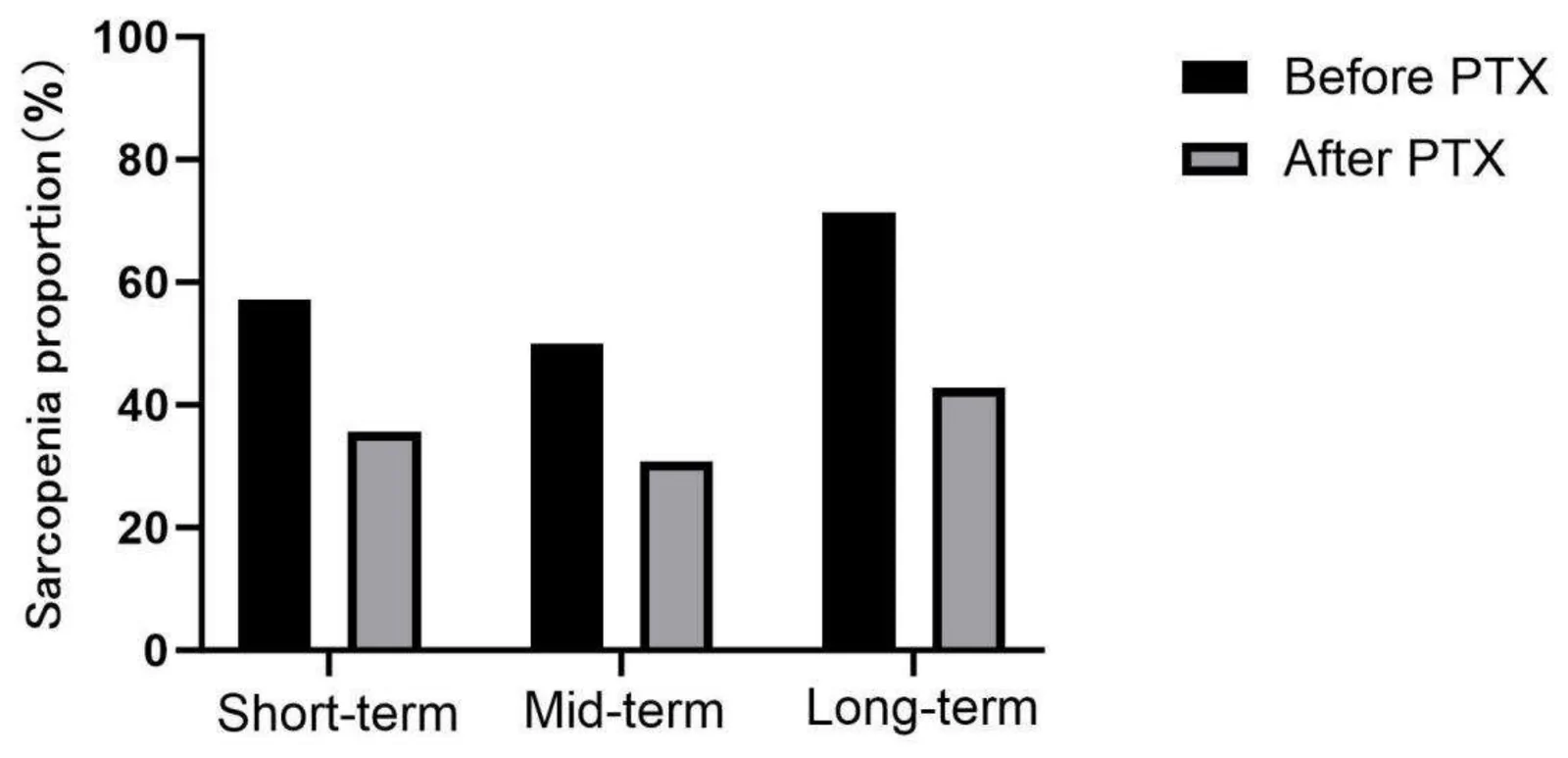
Changes in the proportion of patients with sarcopenia following PTX.

Compared with preoperative levels, the number of patients with sarcopenia showed a consistent numerical downward trend across all postoperative follow-up groups. In the short-term postoperative group, the number decreased from 8 cases (57.1%) preoperatively to 5 cases (35.7%) postoperatively, in the mid-term postoperative group, the number decreased from 13 cases (50%) preoperatively to 8 cases (30.8%) postoperatively, and the long-term postoperative group decreased from 5 cases (71.4%) preoperatively to 3 cases (42.9%) postoperatively. Overall, there was a sustained downward trend in the prevalence of sarcopenia following PTX, indicating that parathyroidectomy may improve sarcopenia in patients with uremia-associated secondary hyperparathyroidism (Figure 6).

Building on this, we further analyzed the dynamic changes in muscle-related parameters at different postoperative stages to clarify the effects of PTX on sarcopenia and the temporal patterns of these effects. 

#### 3.2.1. Short-Term Changes in Muscle-Related Parameters Postoperatively

At 6–12 months after parathyroidectomy (*n* = 14), handgrip strength was significantly higher, while 6-m walking time was markedly shorter than preoperative baseline values, with both differences reaching statistical significance (*p* < 0.05). Lean body mass and the ASMI showed a slight decrease, and skinfold thickness marginally increased relative to the baseline; none of these subtle changes differed significantly (*p* > 0.05) (Table 2).

#### 3.2.2. Mid-Term Changes in Muscle-Related Parameters Postoperatively

At 12–24 months after parathyroidectomy (*n* = 26), lean body mass, the ASMI, and handgrip strength were significantly elevated, whereas the 6-m walking time was markedly reduced relative to the baseline, with significant differences observed (*p* < 0.05). Skinfold thickness increased slightly compared with the baseline, without reaching statistical significance (*p* > 0.05) (Table 3).

### 3.3. Long-Term Changes in Muscle-Related Parameters Postoperatively

At more than 24 months after parathyroidectomy (*n* = 7), skinfold thickness and handgrip strength were significantly elevated, while 6-m walking time was markedly reduced relative to baseline, with all comparisons reaching statistical significance (*p* < 0.05). Lean body mass and ASMI increased marginally versus baseline, with no statistically significant differences observed (*p* > 0.05) (Table 4).

## 4. Discussion

Uremic patients are prone to developing SHPT due to various factors, including the accumulation of uremic toxins, disturbances in calcium and phosphorus metabolism, and chronic inflammation. SHPT further exacerbates muscle damage in these patients, manifesting as reduced muscle mass, weakened muscle strength, and impaired motor function, which severely impacts their quality of life and prognosis [15]. The results of this study indicate that PTX can effectively control SHPT and is associated with a lower sarcopenia prevalence across all postoperative follow-up time points. At all postoperative follow-up time points, patients’ serum PTH levels were significantly lower than preoperative levels, and the number of sarcopenia cases showed a continuous downward trend. This suggests that the surgery not only improves hyperparathyroidism but also correlates with favorable long-term changes in patients’ muscle health.

The results of this study indicate that in the short term following PTX, patients’ muscle strength and motor function showed significant improvement, while indicators related to muscle mass did not show obvious changes. Analyzing the reasons for this, on the one hand, the rapid decline in PTH levels after PTX can quickly alleviate the direct damage caused by high PTH to skeletal muscle cells, which may improve skeletal muscle contractile function [10] and thereby enhance grip strength and shorten walking time. On the other hand, the alleviation of clinical symptoms, such as bone pain after surgery [16], may increase patient activity levels and improve limb coordination, which also enhances motor function. However, the accumulation of muscle mass involves processes such as protein synthesis and the proliferation and differentiation of skeletal muscle cells, which may exhibit a certain degree of lag; therefore, no significant changes were observed in the early postoperative period. Furthermore, lean body mass showed a slight decline 6–12 months postoperatively, which may be related to dietary adjustments in the early postoperative period and changes in protein metabolism under physiological stress; thus, enhanced nutritional support in the early postoperative period is necessary in clinical practice.

The mid-postoperative period emerged as a critical stage for muscle mass improvement, with both lean body mass and the ASMI showing significant increases, suggesting that the improvement in muscle mass following PTX may exhibit a time-dependent effect. During the mid-postoperative period, patients’ PTH levels remained within the normal range, calcium–phosphorus metabolism disorders were continuously corrected, and the body’s “bone-hungry” state was alleviated [17]; simultaneously, improvements in patients’ motor function may have led to increased physical activity, and appropriate exercise can stimulate skeletal muscle protein synthesis, promoting the recovery of muscle mass [18]. However, there were no significant changes in skinfold thickness during this phase, indicating that the increase in muscle mass was not due to fat accumulation but rather a genuine increase in skeletal muscle content, further indicating the specific beneficial effect of PTX on skeletal muscle.

During the long-term postoperative follow-up, patients’ muscle strength and motor function continued to show a trend of significant improvement, and the skinfold thickness increased significantly; however, lean body mass and the ASMI did not show further significant increases. The increase in skinfold thickness suggests that patients’ long-term postoperative nutritional status improved markedly, with increased fat reserves, which may be related to the alleviation of clinical symptoms, improved appetite, and adequate nutritional intake [19]. However, the lack of a sustained improvement in muscle mass indicators may be related to a certain degree of statistical bias due to the small sample size at this stage (*n* = 7), or it may be because uremic patients have irreversible muscle damage caused by toxins, resulting in a certain upper limit to muscle mass recovery [20]. Furthermore, our data indicate sustained improvements in muscle strength and physical function during long-term follow-up. This suggests that sustained favorable muscle strength and physical function indicators remained correlated with PTX status during long-term follow-up, even without further elevations in muscle mass parameters.

In this study, patients’ preoperative PTH levels fell within the typical abnormal range for secondary hyperparathyroidism, providing clear clinical indications for PTX. The postoperative improvement in muscle-related parameters was closely associated with the correction of mineral metabolism disorders, further supporting a link between SHPT and impaired muscle status among uremic patients. In clinical practice, for SHPT patients undergoing PTX, phased clinical intervention strategies should be adopted based on the dynamic changes in muscle parameters at different postoperative stages: In the early postoperative period, the focus should be on enhanced nutritional support, with an appropriate supplementation of high-quality protein and energy to provide an adequate nutritional foundation for skeletal muscle synthesis; in the mid- to long-term postoperative period, while continuing enhanced nutritional interventions, patients should be guided to perform standardized rehabilitation exercises to promote the recovery and remodeling of skeletal muscle mass, optimize mineral metabolism regulation protocols, and sustain improvements in muscle strength and physical function, which may correlate with a better long-term physical status of patients [21].

This study also has certain limitations. As this is a retrospective paired observational study, we did not conduct a continuous multi-time point dynamic follow-up for individual patients and also failed to collect and analyze data on pre- and post-PTX protein intake per kilogram of body weight. In addition, the sample size for the long-term postoperative follow-up was small. Many patients live far away and fail to return for re-examination, resulting in loss to follow-up. This may compromise the statistical power of the results. Additionally, this study did not conduct a multivariate analysis to adjust for potential confounding factors, including age, gender, dialysis vintage, serum albumin and the degree of the PTH reduction. These clinical factors may interfere with muscle mass, strength, and physical function outcomes. Limited by the sample size, further confounding factor corrections could not be performed. Related confounding effects need to be verified in future large-sample studies. These shortcomings make it difficult to fully reflect the long-term trajectory of postoperative muscle-related indicators. Future studies could expand the sample size, conduct multicenter long-term follow-up projects, and monitor the same study subjects at multiple time points to provide a more reliable theoretical basis for developing more targeted clinical intervention plans.

In summary, PTX is associated with sustained improvements in muscle strength and motor function in uremic patients with secondary hyperparathyroidism, and this improvement persists during long-term postoperative follow-up. The improvement in muscle mass exhibits a phased pattern, suggesting that clinicians should implement targeted nutritional and exercise interventions based on the characteristics of different postoperative stages to maximize the beneficial effects of PTX on muscle-related parameters and further enhance patients’ quality of life.

## 5. Conclusions

This study identified phased alterations in muscle-related indicators among uremic patients with SHPT after PTX. PTX was correlated with sustained favorable grip strength and 6-m walking performance across all postoperative stages. Muscle mass increased significantly in the mid-term follow-up period, presenting a time-dependent correlative pattern. The proportion of sarcopenia exhibited a continuous numerical downward trend postoperatively. These findings support stratified nutritional and exercise interventions tailored to different postoperative phases for patients receiving PTX.

## Figures and Tables

**Table 1:** Baseline characteristics of patients.

Characteristics	Short-Term (*n* = 14)	Mid-Term (*n* = 26)	Long-Term (*n* = 7)
Gender [n (%)]			
Male	10 (71.4%)	16 (61.5%)	4 (57.1%)
Female	4 (28.6%)	10 (38.5%)	3 (42.9%)
Age (years)	46.36 ± 9.52	47.96 ± 9.31	41.71 ± 8.48
Dialysis vintage (years)	8.21 ± 3.68	7.81 ± 3.85	6.71 ± 1.50
Dialysis modality, n (%)			
Hemodialysis	14 (100%)	26 (100%)	7 (100%)
Hypertension, n (%)	11 (78.6%)	22 (84.6%)	6 (85.7%)
Diabetes, n (%)	3 (21.4%)	5 (19.2%)	1 (14.3%)
Coronary heart disease, n (%)	1 (7.1%)	3 (11.5%)	1 (14.3%)
Cerebral infarction, n (%)	1 (7.1%)	2 (7.7%)	0 (0%)
HBV infection, n (%)	2 (14.3%)	5 (19.2%)	0 (0%)
Syphilis, n (%)	0 (0%)	0 (0%)	1 (14.3%)
BMI (kg/m^2^)	22.76 ± 3.94	23.07 ± 3.35	20.81 ± 1.49
Hb (g/L)	106.79 ± 13.00	112.00 ± 19.87	103.43 ± 18.77
ALB (g/L)	38.43 ± 3.25	41.08 ± 4.48	41.67 ± 3.47
TC (mmol/L)	3.57 ± 0.59	3.87 ± 1.04	3.45 ± 0.65
TG (mmol/L)	1.36 ± 1.04	2.09 ± 1.64	1.99 ± 1.15
HDL-C (mmol/L)	1.05 ± 0.27	1.10 ± 0.36	1.08 ± 0.33
LDL-C (mmol/L)	2.03 ± 0.53	2.02 ± 0.79	1.65 ± 0.48
Cr (μmol/L)	1019 (814, 1192)	934 (784, 1085)	846 (806, 959)
NT-proBNP (pg/mL)	6574 (2644, 26,930)	5075 (1520, 17,366)	2078 (1058, 2854)
Troponin (ng/mL)	0.06 (0.04, 0.09)	0.04 (0.03, 0.06)	0.03 (0.02, 0.05)
PTH (pg/mL)	1479 (832, 1730)	1103 (888, 1657)	1188 (806, 2110)
Serum phosphorus (mmol/L)	2.55 ± 0.51	2.44 ± 0.48	2.10 ± 0.56
Serum calcium (mmol/L)	2.46 (2.22, 2.66)	2.59 (2.41, 2.65)	2.43 (2.19, 2.49)
ALP (U/L)	228 (124, 541)	201 (130, 344)	268 (136, 867)

**Table 2:** Comparison of muscle-related parameters before surgery and 6–12 months after surgery.

Time Point	Lean Body Mass (kg)	ASMI (kg/m^2^)	Skinfold Thickness (mm)	Grip Strength (kg)	6 m Walking Time (s)
Preoperative	43.53 ± 9.66	6.40 ± 1.40	16.32 ± 2.77	26.67 ± 4.33	5.47 ± 0.72
6–12 months postoperatively	42.32 ± 9.72	6.32 ± 1.39	16.50 ± 2.83	28.10 ± 4.81	5.28 ± 0.64
Percentage change relative to baseline	−2.47% ± 10.12% #	−0.15% ± 12.77% #	+1.48% ± 10.69% #	+5.20% ± 4.57% *	−3.24% ± 3.56% *

*n* = 14, # *p* > 0.05, * *p* < 0.05.

**Table 3:** Comparison of muscle-related parameters before surgery and 12–24 months after surgery.

Time Point	Lean Body Mass (kg)	ASMI (kg/m^2^)	Skinfold Thickness (mm)	Grip Strength (kg)	6 m Walking Time (s)
Preoperative	41.95 ± 9.26	6.23 ± 1.10	15.27 ± 4.18	26.25 ± 6.38	5.83 ± 0.90
12–24 months postoperatively	44.12 ± 7.74	6.74 ± 0.94	15.88 ± 3.31	27.28 ± 5.79	5.44 ± 0.79
Percentage change relative to baseline	+6.46% ± 11.03% *	+9.46% ± 13.49% *	+6.19% ± 11.73% #	+5.02% ± 9.34% *	−6.18% ± 8.36% *

*n* = 26, # *p* > 0.05, * *p* < 0.05.

**Table 4:** Comparison of muscle-related parameters before surgery and over 24 months after surgery.

Time Point	Lean Body Mass (kg)	ASMI (kg/m^2^)	Skinfold Thickness (mm)	Grip Strength (kg)	6 m Walking Time (s)
Preoperative	39.47 ± 6.34	5.91 ± 0.70	16.07 ± 2.34	24.56 ± 5.31	6.13 ± 1.05
>24 months postoperatively	42.35 ± 8.17	6.26 ± 0.96	17.43 ± 2.30	25.91 ± 5.01	5.49 ± 1.12
Percentage change relative to baseline	+7.55% ± 15.10% #	+6.05% ± 12.91% #	+8.68% ± 3.29% *	+6.19% ± 5.85% *	−10.29% ± 9.94% *

*n* = 7, # *p* > 0.05, * *p* < 0.05.

## Data Availability

The data presented in this study are not publicly available due to patient confidentiality and ethical restrictions. The dataset was collected by the authors at our clinic and is stored in institutional records. Data may be made available from the corresponding author upon reasonable request, subject to appropriate ethical approval and institutional permissions.
